# Knowledge Ecology and Policy Governance of Green Finance in China—Evidence from 2469 Studies

**DOI:** 10.3390/ijerph20010202

**Published:** 2022-12-23

**Authors:** Junjie Li, Bei Zhang, Xin Dai, Meng Qi, Bangfan Liu

**Affiliations:** 1 School of Public Administration, Yanshan University, Qinhuangdao 066004, China; 2 Hebei Public Policy Evaluation and Research Center, Qinhuangdao 066004, China; 3 Institute of Marxism, Shandong University, Jinan 250100, China; 4 Institute of Marxism, Chinese Academy of Social Sciences, Beijing 300712, China

**Keywords:** China, green finance, ecology of knowledge, public policy

## Abstract

CiteSpace was used to visualize the knowledge ecology of the green finance research literature in CNKI and WOS, and NVivo software was used to root the code analysis of the current green finance policies in China. From the analysis of the research hotspots, both in China and internationally, great importance is attached to the research on green finance, and the research on green financialization has broad prospects. The core group of authors on green finance research in China has taken shape, whereas the core group of authors of green finance research in the rest of the world has not yet taken shape. There is a lack of close cooperation and a relatively low level of communication among important domestic green finance research institutions, and a certain scale of cooperation network has been formed among influential international institutions. The major countries for influential international green finance research are Singapore, France, Switzerland, Canada and Saudi Arabia, and the international influence of China’s green finance needs to be improved. Both domestic and foreign countries attach great importance to the balance between economic growth and the low-carbon green transition. China attaches more importance to macroeconomic development and strategic transition, but internationally, the trend is toward microcorporate green performance, policy optimization and market innovation. The research focus of green finance has achieved in three stages of evolution, namely, green industry in the early stage, green services in the middle stage and green strategy in the near future. International green finance research focuses on climate change, market players, government performance, social responsibility sharing, etc. In particular, reducing the cost of green development is the focus of international green finance. The domestic focus is on climate risk, carbon neutrality, carbon peak, low-carbon transition, carbon reduction, and green transition themes. Internationally, the focus is on financial performance, decisions, green finance, credit, drivers, quality, socially responsible investment and other topics. Considering the practical implementation of green finance in China, the governance logic of China’s green finance policy consists of five main categories: policy belief, policy objective, policy tool, policy feedback and policy cycle. In the future, the development and improvement of China’s green finance policy should achieve breakthroughs in the following aspects: first, guiding the main body of green finance policy to firmly establish policy beliefs; second, improving the clarity of green finance policy objectives; third, enhancing the overall effectiveness of the governance of green financial policy instruments; fourth, strengthening the green finance incentive policy feedback system construction; and fifth, improving the quality of green finance policy cycles.

## 1. Introduction

Green finance generally refers to all financial integration activities and processes that give consideration to human economic–social development and ecological environmental protection. Green finance plays a significant role in promoting economic growth and provides important support for national green transformation development. Green finance has the advantages of cost sharing and risk sharing. The effective coordination between green finance policy and green fiscal policy is an effective means to promote high-quality development. In today’s world, the global climate change situation is severe. Green development has become the inevitable choice for the development of human society, and an increasing number of green development issues have come into people’s view. From a political and policy perspective, green finance has become highly recognized around the world. The implementation of green finance has not only won political recognition from countries, regions, politicians and entrepreneurs but also from the general public.

In recent years, with the proposal of China’s goal of achieving a carbon peak and carbon neutrality, the realization of green, low-carbon and high-quality development has become an internal requirement of China’s development. In this context, green finance has attracted much attention from domestic scholars. Scholars have focused on green finance (539, number of documents, similarly below), green credit (115), green bonds (76), carbon neutrality (51), green development (41), commercial banks (40), green finance and the financial system (38), high-quality development (31), rural revitalization (29), financial reform and innovation (28), the Belt and Road Initiative (26), green finance policy (25) and carbon finance (22). There is rich theoretical research that has been carried out on such topics as high-quality economic development (21), green credit policy (21), environmental information disclosure (19), green transformation (18) and Chinese commercial banks (18). There is also research on finance (1471, number of supporting documents, the same below), environmental science and resource utilization (647), investment (333), economic system reform (326), economic theory and history of economic thought (314), securities (224), macroeconomic management and sustainable development (143), enterprise economics (104), agricultural economics (86), industrial economics (70), meteorology (33), economic law (27), trade economics (26), power engineering (23), finance and taxation (22), accounting (21), information economy and postal economy (19), administrative law and local regulations (17), insurance (17), market research and information (13) and other disciplines. The research also includes development research–policy research (64, number of supporting literature, similarly below), applied research–management research (21), engineering research (19), applied research–policy research (17), development research–industry research (16), applied research (15), applied research–industry research (10), development research–management research (9), development research (7) and other research levels. Among them, the top 50 authoritative studies cited in the CNKI database are mainly (in descending order of citation frequency) “On Building China’s Green Finance System” [1]; “Refinancing Environmental Verification, Environmental Information Disclosure and Equity Capital Cost” [2]; “Does Green Credit Affect the Investment and Financing Behavior of Heavily Polluting Enterprises?” [3]; “The Mechanism of Financial Development Affecting Regional Green Development: A Study Based on Ecological Efficiency and Spatial Metrology” [4]; “Green Finance and Sustainable Development” [5]; “The Logic and Framework of Developing China’s Green Finance” [6]; “The Impact of Green Credit on the Credit Risk of Commercial Banks” [7]; “Comparative Study on the Development of Green Bonds in China and the Standards between China and Foreign Countries” [8]; “The Connotation, Mechanism and Practice of Green Finance” [9]; “Green Finance Policy, Corporate Governance and Corporate Environmental Information Disclosure: A Case Study of 502 Listed Companies in Heavily Polluting Industries” [10]; “Comparative Study on Green Finance Products at Home and Abroad” [11]; “Green Finance Development and Innovation Research” [12]; “Green Credit, Internal and External Policies and the Competitiveness of Commercial Banks: An Empirical Study based on 9 Listed Commercial Banks” [13]; “International Green Bond Market: Current Situation, Experience and Enlightenment” [14]; Study on the Contribution of Green Finance to China’s Economic Development” [15]; “Some Thoughts on the Transformation from Traditional Finance to Green Finance” [16]; “Review of Green Finance Research” [17]; “How can Green Credit Policies be Effectively Implemented by Commercial Banks?—Research based on Evolutionary Game Theory and DID Model” [18]; “Based on Joint Analysis of the Influence Factors of Green Finance” [19]; “The Development and Prospect of Green Finance in China” [20]; “Should China’s Financial Institutions Bear Environmental Responsibility?—Basic Facts, Theoretical Models and Empirical Tests” [21]; “’Green Finance’ and Sustainable Economic Development” [22]; “Research and Practice of Finance Promoting Sustainable Development” [23]; “An Empirical Analysis of the Impact of Green Credit on the Upgrading of China’s Industrial Structure: Based on China’s Provincial Panel Data” [24]; “Build a Green Financial Service System Marked by ‘Carbon Finance’” [25]; “Exploration on the Long-Term Mechanism of Sustainable Development of Green Finance in our Country” [26]; “Development Trend and Risk Characteristics of International Green Bonds” [27]; “Green Bond Theory and Analysis of Chinese Market Development” [28]; “Green Finance, Social Responsibility and Behavior Choice of State-Owned Commercial Banks” [29]; “Positive Analysis of Green Credit to our Industrial Structure Optimization Effect” [30]; “Green Credit Policy Promotes Green Innovation Research” [31]; “The Impact of Green Credit on the Performance and Risk of Commercial Banks: Based on the Panel Data Analysis of 16 Listed Commercial Banks” [32]; “Research on Incentive Policy of Green Credit based on DSGE Model” [33]; “On the ‘Green Revolution’ of Finance” [34]; “Investment and Financing Problems and Mechanism Innovation of Environmental Protection Industry in China” [35]; “Ecological Product Connotation and its Value Realization Way” [36]; “Current Situation and Prospect of Third-Party Green Bond Certification in China” [37]; “Green Finance System in China: Construction and Development Strategy” [38]; “Can Green Credit Promote Technological Innovation of Environmental Protection Enterprises” [39]; “Study on the Operation Mechanism of Green Credit in China’s Commercial Banks” [40]; “Our Green Finance Development Present Situation and Policy Suggestion” [41]; “Practice and System Innovation in the Development of Green Finance” [42]; ”Does Green Credit Policy Pay off or Pay Off?—Cost-Efficiency Analysis of PSM-DID~1 from the Perspective of Resource Allocation” [43]; “Measurement and Comparison of the Development Level and Efficiency of Green Finance in China—Based on the Micro Data of 1040 Public Companies” [44]; “’Green’ Policies and Allocative Efficiency of Green Finance: An Empirical Study of Listed Manufacturing Companies in China” [45]; “Research on the Relationship between Green Credit Scale and the Competitiveness of Commercial Banks” [46]; “Green Finance Development, Debt Maturity Structure and Green Enterprise Investment” [47]; “Rural Financial Dilemma and Innovative Choice based on the Perspective of Rural Revitalization” [48]; “The Impact of Green Credit on the Business Performance of Commercial Banks—Based on the Empirical Analysis of Chinese Commercial Banks” [49]; “On the Legalization of Green Credit Policy” [50]. 

In summary, in view of the externality of environmental protection and the profit-driven nature of finance itself, the importance of policies for the development of green finance has become an academic consensus. At present, there are abundant research achievements in theoretical analysis, practical exploration, policy optimization, framework interpretation and other aspects of green finance, but the research on the combination of macro green finance policy theory and policy texts, especially the interpretation of theoretical models, is still lacking and needs to be studied and strengthened. Therefore, this study analyzes the above problems, and from theory to practice, it is a work of certain value.

## 2. Research Design

### 2.1. Research Methods and Innovation Points

In this paper, CiteSpace software was used for the bibliometric analysis and the atlas analysis. CiteSpace5.8R3 was mainly used to draw the knowledge map, and the hotspots, frontiers, core authors and cooperative networks of green finance were visualized and analyzed [51,52,53,54]. This study used Nvivo12plus to conduct the content analysis of the policy texts [55], mainly aiming at China’s current green finance policy texts, hoping to obtain the structure, system and operation of China’s green finance policy and the main framework, main content and basic logic.

The dialectical relationship between the bibliometric analysis and grounded theory research is mainly reflected in the following two aspects: On the one hand, the bibliometric analysis summarizes the research on green finance in general, providing a reference for the generation of the three-level rooted coding category. Based on the science-based econometric analysis, it minimizes the influence of subjectivity on coding. On the other hand, the grounded theoretical modeling unifies the conclusions drawn from the bibliometric analysis with the policy text, further abstracts the governance framework and rules of our green financial policy from the perspective of policy science and realizes the layer deepening of the study of green financial policy from the research literature to the policy text and theoretical modeling. In a word, the bibliometric analysis is the foundation and bedding of the theoretical research, and the grounded theoretical research is the deepening and improvement of the bibliometric analysis.

The main innovation points of this paper are as follows: First is the innovation of the research perspective. From the perspective of policy science, this paper examined the basic logic of Chinese green finance policy research and policy governance, hoping to put forward countermeasures and suggestions for our green finance policy to better support and serve our green finance development. The second is the innovation of research methods. In this paper, the bibliometric analysis and grounded theory analysis methods were mixed to clarify the shortcomings and improvement directions of green finance policies from the intersection of theory and policy. The third is the innovation of research theory. In terms of the theoretical research and practice of China’s green finance, China’s green finance pays attention to the macroscopic theoretical guidance and strengthens the implementation of projects. This mainstream thinking of combining theory and practice is conducive to enriching and deepening the guiding and instrumental nature of green finance research and has enlightening significance for relevant studies at home and abroad.

### 2.2. Data Sources

Domestically, Yu conducted the accurate retrieval of academic journals in the CNKI database with “topic = green finance”. In order to ensure the authoritativeness and representativeness of the text collection, the source category was set as Peking University Core and CSSCI. In total, 1729 studies were retrieved. Excluding magazine introductions, calls for papers, news, book reviews, key topic selections, interpretations, book recommendations and other literature types, 1655 effective research texts were retained. On the international front, in the Web of Science Core Collection database, “TS = (“green financ*”) OR TS = (“environment* finance”) OR TS = (“sustainable finance”) OR TS = (“green banking”)” was used as the search condition for the advanced search. To ensure the authoritativeness and representativeness of the text collection, this paper selected the SSCI and SCI databases and retrieved a total of 896 texts. After manual screening, 792 effective research texts were retained.

### 2.3. Study Process

**Figure 1:**
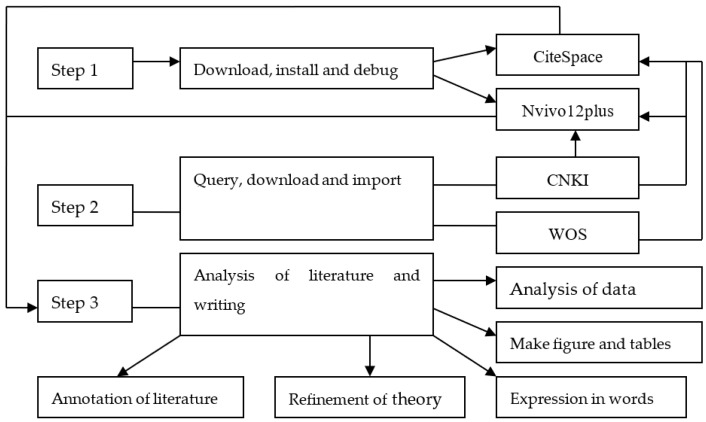
Flow chart of the study [56].

The research process of this study mainly included three steps: First, download the software and install and debug it. This included downloading CiteSpace and Nvivo12plus and installing and debugging them. Second, search, download the research data and import in the software. This included searching and downloading the research data from CNKI and WOS and importing them into the software. Thirdly, analyze the literature and write the papers. This mainly included the data analysis, chart making, text expression, theory extraction and literature annotation. The entire process is represented in Figure 1.

## 3. Analysis of the Knowledge Ecology

### 3.1. Publication Analysis

Publication analysis is the process of decomposing and analyzing the quantity, trends and composition of the published literature in a research field from different dimensions. By analyzing the number of published papers in a research field, researchers can grasp the total number of published papers in the research field, the annual number of published papers and predict the trends in the published papers.

**Figure 2:**
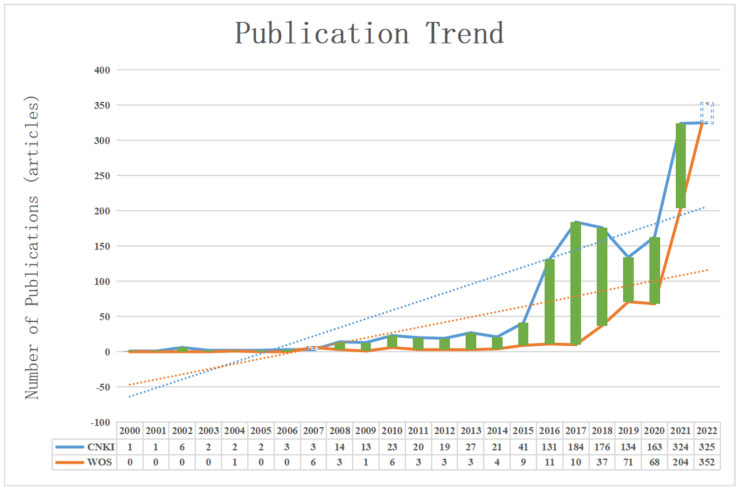
Trends in the publications.

A publication trend chart was drawn based on the number of publications in the CNKI and WOS databases (Figure 2). As can be seen from Figure 2, in terms of the earliest publication date and the total number of publications, domestic studies on green finance began in 2000. From 2000 to the retrieval date, the CNKI database excluded studies in lost years, and 1635 related studies in total were published. The international literature on policy and environment began to be published in 2004. Since 2004, the WOS database has published 792 related studies. In terms of the overall release trend (as shown by the dotted line in the figure), the number of releases in both international and domestic policy environments is on the rise. In terms of the number of published papers, the average annual number of published papers is 71.09 domestically and 41.68 internationally. On the whole, both domestic and international researchers attach great importance to green finance. From the trend in the publications, the development speed of green finance in China is faster than that of the world. Overall, the green finance research is promising.

### 3.2. Analysis of the Research Subjects and Cooperation Network

Today’s progress on the study of green finance cannot be achieved without the unremitting efforts of relevant researchers and teams. Through an analysis of the structural characteristics of the authors and their cooperative networks, the core group of authors and their cooperative relationships in this field can be reflected.

Core authors refer to scholars with a higher academic level and more scientific achievements in a certain field. Analyzing the core authors is helpful for understanding the research status and progress of this field. According to Price’s law, the number of core authors’ publications can be calculated as follows:

$$\mathrm{MP}=0.749\sqrt{NP\max}$$

where MP represents the minimum number of the published papers by the core authors, and *NP*max represents the cumulative number of the published papers by the authors with the most papers within the research period. If a stable core group of authors accounts for 50% of the total number of papers, it can be considered that a core group of authors has been formed in this field.

**Figure 3:**
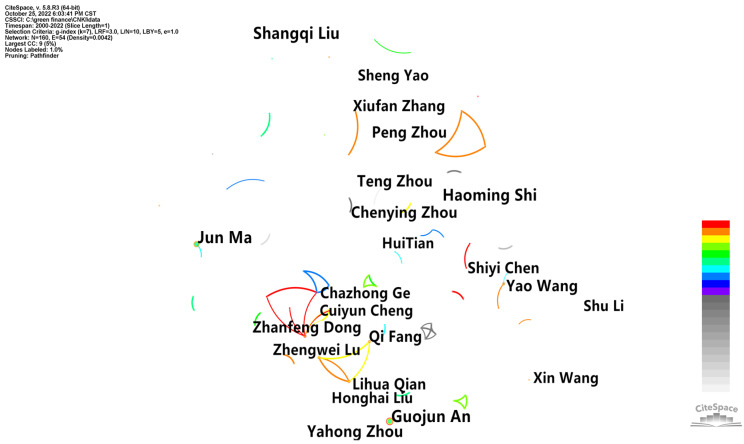
Domestic author cooperation chart.

**Figure 4:**
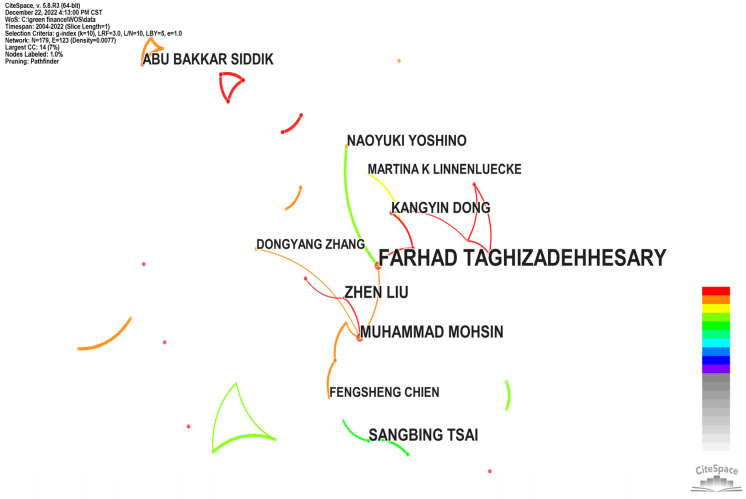
International author collaboration chart.

In the domestic aspect, the retrieval information of 1655 studies was imported into CiteSpace for the visualization analysis of the authors’ status. We obtained the number of articles published by domestic authors (Table 1) and the cooperation (Figure 3 and Figure 4). As shown in Table 1, “Count” represents the number of articles published by the corresponding author, and “Year” represents the number of years in which the author first published articles. It can be found that two scholars, Jun Ma and Guojun An, published the most articles, with 17 articles each. Shangqi Liu, the author, followed with 16 articles, Haoming Shi with 15 articles and Yahong Zhou with 13 articles. According to the calculation formula, the MP value was 3.08; that is, one could be regarded as a core author in the field of green finance research in China with four or more papers. According to CiteSpace, it was found that there were 32 core authors of domestic green finance research, and 10 are listed in Table 1. The other 22 were Zhanfeng Dong (9), Peng Zhou (9), Xiufan Zhang (8), Zhengwei Lu (8), Chazhong Ge (7), Shiyi Chen (6), Cuiyun Cheng (5), Hui Tian (5), Sheng Yao (5), Shu Li (5), Xin Wang (5), Honghai Liu (5), Weimin Ouyang (4), Mingdi Cao (4), Feng Xu (4), Wen Wang (4), Yueqiu Zhou (4), Wei-An Li (4), Kang-shi Wang (4), Feng-Rong Wang (4), Xian-Ming Duan (4) and Yu Qi (4). In general, in terms of the number of papers published, the outstanding authors, represented by Jun Ma, Guojun An, Shangqi Liu, Haoming Shi and Yahong Zhou, have formed in the field of green finance research. Among them, Shangqi Liu, Haoming Shi, Yahong Zhou, Teng Zhou and Chenying Zhou all began to pay attention to green finance in 2000, indicating that the above core authors have been paying attention to green finance for more than 20 years and have formed a series of achievements. Jun Ma, Guojun An, Yao Wang, Qi Fang and Lihua Qian began to pay attention to green finance after 2015, and Jun Ma and Guojun An started to publish the first number of articles in 2016, indicating that Jun Ma and Guojun An are the authors who started to pay attention to green finance in recent years and have produced more high-quality achievements. According to the statistics on the authors, the core authors have published 249 articles in total, accounting for 50.72% of the total number of policy and environmental research studies, which is higher than the standard of core authors, indicating that the core authors of domestic green finance research have initially formed.

From an international aspect, the retrieval information on 792 studies was imported into CiteSpace for the visualization analysis of the authors’ status, and it was found that Farhad Taghizadehhesary published the most papers on green finance in WOS, with 15 papers, which were calculated according to the calculation formula. The MP value obtained was 2.90; that is, authors with three or more publications can be regarded as core authors in the field of international green finance research. There were 29 core authors in the field of international policy and environment studies, and only the top 10 are listed in Table 1. In addition, the other 19 were Dongyang Zhang, Lin Lin, Dilvin Taskin, Dariusz Wojcik, Jianchao Luo, Wasim Iqbal, Xiucheng Dong and Chienchiang Lee, Arshad Ahmad Khan, Shanshan Li, Muhammad Sadiq, Quangthanh Ngo, Kai Wang, Xianchun Liao, Juri Hinz, Muhammad Irfan, Eyup Dogan, Muhammad Abu Sufyan Ali and Shanglei Chai. Except for Dongyang Zhang, who published four papers, the others had three papers. According to the statistics on the authors, the number of studies published by the core authors was 119 articles, and the number of papers published by them accounted for 37.30% of the total number of international green finance research papers, which is lower than the number of papers published by the core authors, indicating that the core authors of international green finance research have not yet formed.

**Figure 5:**
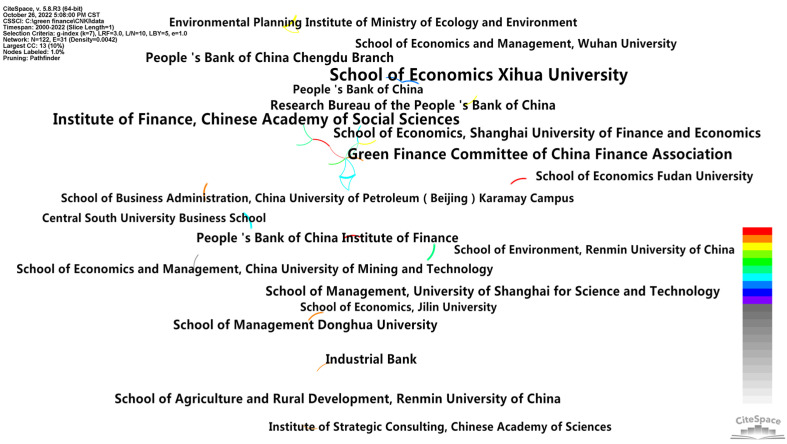
Cooperation network of the domestic institutions.

**Figure 6:**
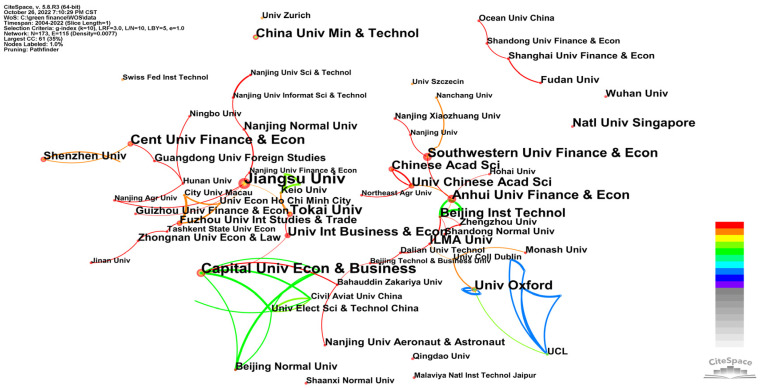
Network of the international institutions.

In order to explore the cooperation of research institutions in the field of green finance, a co-occurrence analysis was conducted on the signed institutions of the sample literature, and an institutional cooperation network map was obtained (Figure 5 and Figure 6). The size of the tree rings was proportional to the number of publications, and the lines between the nodes and their thickness represent the cooperation relationship and cooperation frequency between institutions. As shown in Figure 5, the number of nodes in the cooperative network of the domestic research institutions was N = 122; the number of connections was E = 31; and the network density was D = 0.0042. In other words, 122 institutions and 31 links between the institutions were selected in the knowledge map of green finance research in China. In terms of the number of articles published by institutions (see Table 2), the School of Economics of Xihua University published the most papers, with 43 papers, followed by the Institute of Finance and Banking of the Chinese Academy of Social Sciences, with 32 papers and the Green Finance Committee of the Chinese Society of Finance and Banking, with 29 papers. As can be seen from Figure 5, the connections were less than nodes, and the density was relatively low, which indicates the lack of close cooperation between the institutions with important contributions to this field, and the degree of communication was relatively low. Thus, all institutions should strengthen cooperation, exchange theory frontiers and promote research development in the future. As shown in Figure 6, the number of nodes in the cooperation network of the international research institutions was N = 173; the connection was E = 115; and the network density was D = 0.0077. In other words, 173 institutions and 115 links between the institutions were selected from the map of the knowledge in the field of international policy and environmental research. In terms of the number of publications published by the institutions (see Table 2), Jiangsu University had the most publications, with 19 papers, followed by Capital University of Economics and Business, with 15 papers and then Tokai University, with 13 papers. On the whole, compared with China, the number of publications published by influential international institutions was relatively small; however, the close connection between the influential international institutions formed at a certain scale a cooperation network.

**Figure 7:**
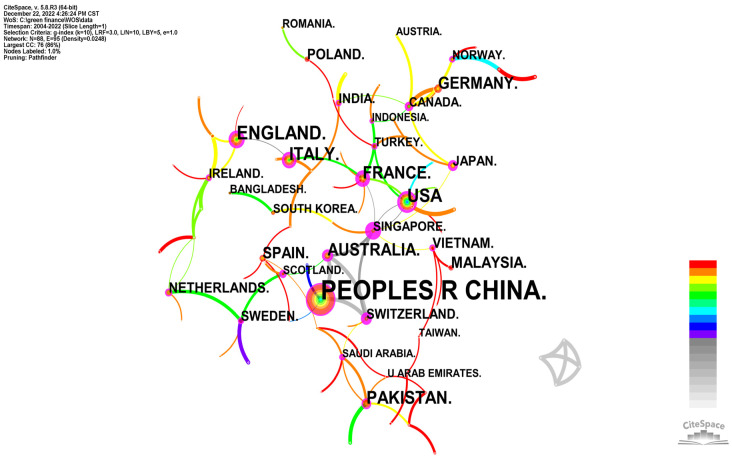
Map of the national cooperation networks.

By analyzing the publication status, cooperation network and publication trends of different countries in a research field, researchers can quickly grasp the national importance, cooperation status, publication trends and national influence. In order to explore the cooperation of research institutions in the field of policy and environment research, a co-occurrence analysis of the signed countries of the sample literature was carried out, and a comparison table of the number and centrality of the national publications (see Table 3) and a map of the national cooperation network (Figure 7) were obtained. The size of the tree rings was proportional to the number of publications, and the lines between the nodes and their thickness represent the cooperation relationship between the institutions and the frequency of the cooperation.

As shown in Figure 7, the number of nodes in the national cooperative network was N = 88, the connection was E = 95, and the network density was D = 0.0248. In other words, 88 countries and 95 links between countries were selected from the knowledge map of green finance research fields in different countries, indicating that there are a large number of countries concerned about green finance in the world, and there is a relatively close cooperation between countries. In terms of the number of articles published by countries (see Table 3), the top five countries were the People’s Republic of China (417 articles published), the United Kingdom (70 articles published), the United States (61 articles published), Italy (54 articles published) and Pakistan (51 articles published). This shows that scholars in the above countries pay more attention to green finance, and a large number of academic achievements have been published. In terms of national intermediation (see Table 3), the top five countries in terms of centrality were Singapore (0.91), France (0.51), Switzerland (0.4), Canada (0.35) and Saudi Arabia (0.32). This shows that Singapore, France, Switzerland, Canada and Saudi Arabia are relatively influential countries in the field of green finance. Overall, the United States ranks among the top seven countries in terms of both the number of publications and the centrality, indicating that the United States has a greater influence in the field of green finance. In terms of this document, our country has attached great importance to green finance since 2008, but from a central point of view, our international influence in the field of green finance still needs to be improved.

### 3.3. Analysis of the Research Hotspots

#### 3.3.1. Keywords in the Co-Occurrence Analysis

Keywords are words that an author refines to summarize the topic of an article as well as the author’s highly summarized and refined academic thought, research theme and research content in a specific study. Therefore, keywords can also become an approach and method to analyze a research topic. At the same time, the areas of interest in the research in this field can be understood by examining the frequency of keywords, and the speed of updates and the research vitality in this field can be judged.

**Figure 8:**
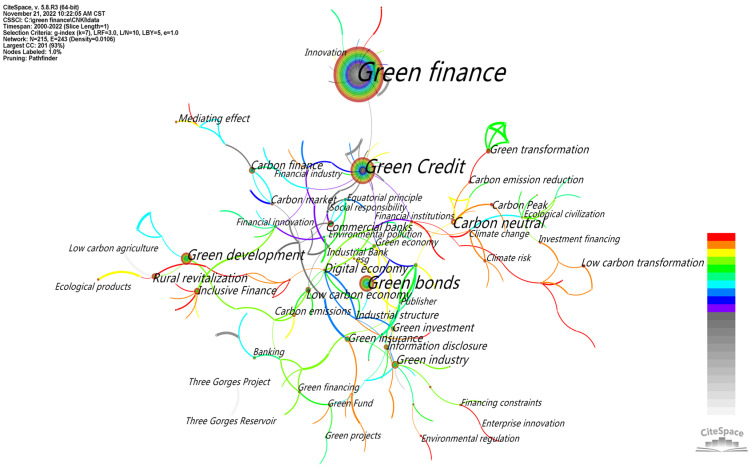
CNKI keyword co-occurrence.

**Figure 9:**
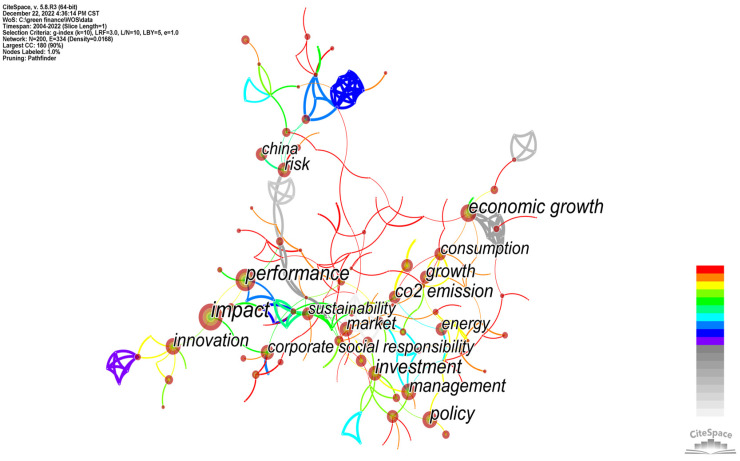
WOS keyword co-occurrence.

In an analysis of the knowledge graph, the research topics and key areas of interest of a certain leader can be obtained by a keyword analysis. In the network image of keyword co-occurrence constructed using CiteSpace software, each node represents a keyword, and the size of the node represents the frequency of the keyword’s occurrence. CiteSpace was run, the node type was set as the keyword and the time range was set as 2000–2022 for domestic and 2004–2022 for international. The research data were imported, and the keyword co-occurrence graph was obtained (Figure 8 and Figure 9). The keyword frequency and centrality diagram table (see Table 4) was by using the “keyword” function in CiteSpace. As can be seen from the figure, there were altogether 215 domestic nodes with 243 connected nodes (density = 0.0106) and 200 international nodes with 334 connected nodes (density = 0.0168). Domestically, as shown in Table 4, the top keywords were green finance (1021), green credit (267), green bond (188), carbon neutrality (102), green development (95), digital economy (48), rural revitalization (48), green industry (45), low-carbon economy (45) and carbon finance (42). This shows that the domestic research on green finance mainly focuses on green financial products, green transformation development strategy and green (low-carbon) industry and carbon economy and the coupling relationship between them. Internationally, as shown in Table 4, the top keywords were impact (129), performance (95), investment (80), economic growth (76), policy (70) and CO2 emissions (60), growth (54), management (53), innovation (52) and market (49). This shows that the international research on green finance mainly focuses on the impact of climate change, corporate performance, investment, economic growth, policy intervention, carbon dioxide emissions, market innovation and management, etc. Above all, we can see that both domestic and international researchers attach great importance to balancing the relationship between economic growth and low-carbon green transition. However, Chinese green finance research focuses more on macroeconomic development and strategic transition, while the international community tends towards microcorporate green performance, policy optimization and market innovation.

#### 3.3.2. Keyword Cluster Analysis

**Figure 10:**
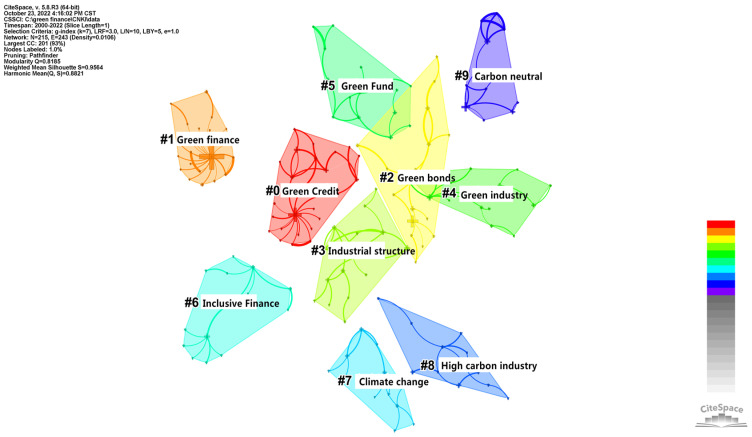
CNKI keyword clustering.

**Figure 11:**
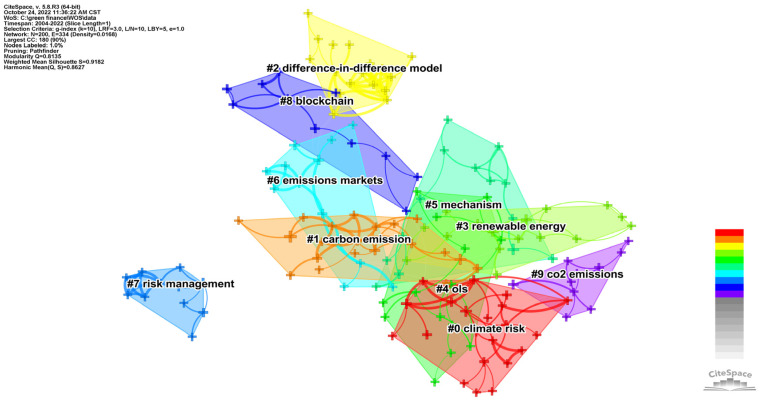
WOS keyword clustering.

CiteSpace measures the effect of mapping by the Q value and S value according to the network structure and clarity of the clustering. First, the value of Q was modularity, meaning a module value with an interval of [0, 1]. Q > 0.3 means that the divided community structure was significant. The second value was S-weighted mean silhouette, which stands for the average silhouette values. S > 0.5 indicates that the clustering was reasonable, and S > 0.7 indicates that the clustering was efficient and convincing. From a domestic aspect, as shown in Figure 10, the Q value of the keyword clustering graph was 0.8185; thus, its structure was significant. Its S value was 0.9564. From an international aspect, as shown in Figure 11, the Q value of the keyword clustering graph was 0.8135, and the S value was 0.9182. It was not only larger than the reasonable average contour value of 0.5 but also larger than 0.7, indicating that the cluster analysis in this paper was efficient and convincing. Cluster results with a significant structure and good effect are helpful for us to analyze and grasp the overall characteristics and development trends of policy environment research. As shown in Figure 10, the top eight clusters in China were “#0 green credit”, “#1 green finance”, “#2 green bonds”, “#3 industrial structure”, “#4 green industry”, “#5 green fund”, “#6 inclusive finance”, “#7 climate change” and “#8 high-carbon industry”. As shown in Figure 11, the top ten clusters in the international rankings were “#0 climate risk”, “#1 carbon emission”, “#2 difference-in-difference model”, “#3 renewable energy”, “#4 ols”, “#5 mechanism”, “#6 emissions markets”, “#7 risk management”,“#8 blockchain” and “#9 co2 emissions”.

The keyword cluster summary table was drawn to further probe the number of keywords contained in each cluster, the tightness of the cluster itself, the average year of keyword distribution and the main keywords contained in the cluster. As can be seen in Table 5, Figure 10 and Figure 11, the mean silhouette (S-value) of the clustering was above 0.7, indicating that each clustering was efficient and convincing. Domestically, the number of nodes contained in each cluster: Cluster 0 (green credit)—25 nodes; Cluster 1 (green finance)—23 nodes; Cluster 2 (green bonds)—16 nodes; Cluster 3 (industrial structure)—16 nodes; Cluster 4 (green industry) and Cluster 5 (green Fund)—15 nodes each; Cluster 6 (inclusive finance)—14 nodes; Cluster 7 (climate change)—13 nodes; Cluster 8 (high-carbon industry) and Cluster 9 (carbon neutral)—12 nodes each. Internationally, the number of nodes included in each cluster: Cluster 0 (climate risk)—21 nodes; Cluster 1 (carbon emission)—20 nodes; Cluster 2 (difference-in-difference model)—17 nodes; Cluster 3 (renewable energy)—16 nodes; Cluster 4 (ols)—15 nodes; Cluster 5 (mechanism)—14 nodes; Cluster 6 (emissions markets)—13 nodes; Cluster 7 (risk management) and Cluster 8 (block chain)—11 nodes; and Cluster 9 (co2 emissions)—10 nodes. The keywords extracted according to the weighted algorithm are listed in each cluster, and the top five in each cluster are ranked from left to right in order of importance from the largest to the smallest.

#### 3.3.3. Keyword Time Sequence Analysis

##### Timeline Analysis

**Figure 12:**
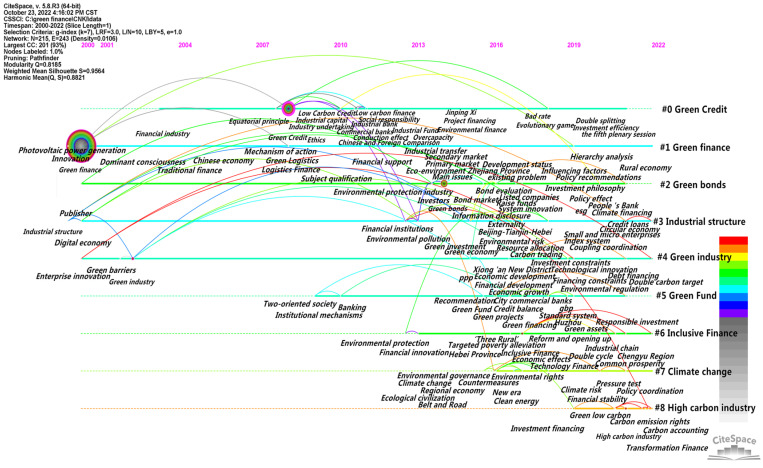
CNKI timeline.

**Figure 13:**
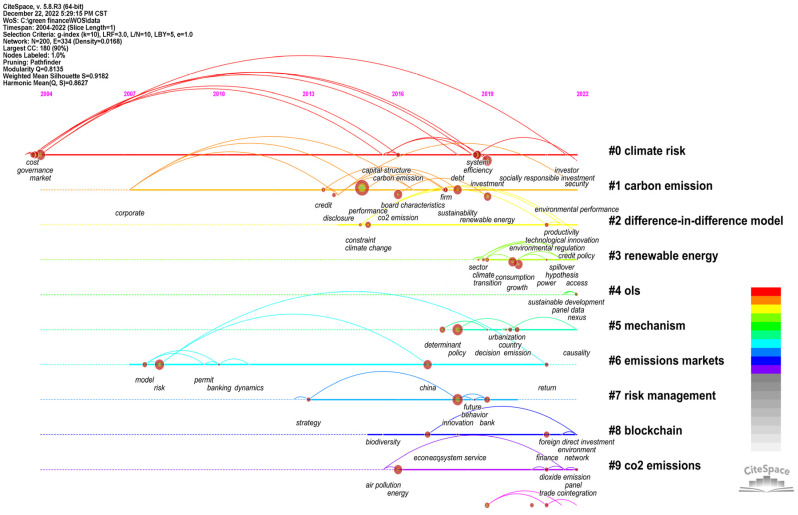
WOS timeline.

CiteSpace software was used to draw the timeline map of the keywords in the field of policy environment, and the duration and evolution trend of research hotspots could be obtained, as shown in the figure. The keyword evolution map is arranged chronologically from left to right, and the size of the circular nodes in the graph is proportional to the occurrence frequency of the corresponding keywords. CiteSpace software was used to analyze the evolution of topics of interest in the selected literature. In domestic terms, the time parameter was set as 2000–2022, year per slice = 1, g-index was k = 7, and node type = keyword. The running results of the keyword clustering evolution are shown in Figure 12. In total, 215 nodes and 243 lines were generated in the obtained atlas. At this time, Q = 0.8185, indicating that the structure of the division was significant. Internationally, the time parameter was set as 2004–2022, year per slice = 1, g-index was k = 10, and node type = keyword. The running results of the inscription clustering evolution is shown in Figure 13. In total200 nodes and 334 lines were generated in the obtained international atlas. At this time, Q = 0.8135, indicating that the structure of the division was significant.

##### Time Zone Analysis

**Figure 14:**
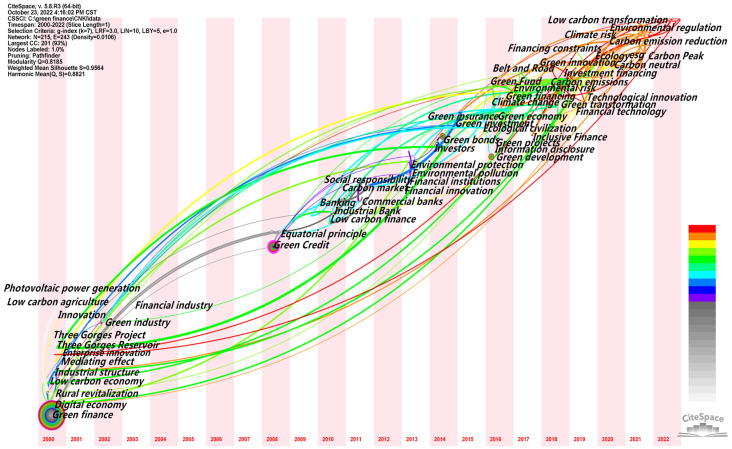
CNKI time zone chart.

**Figure 15:**
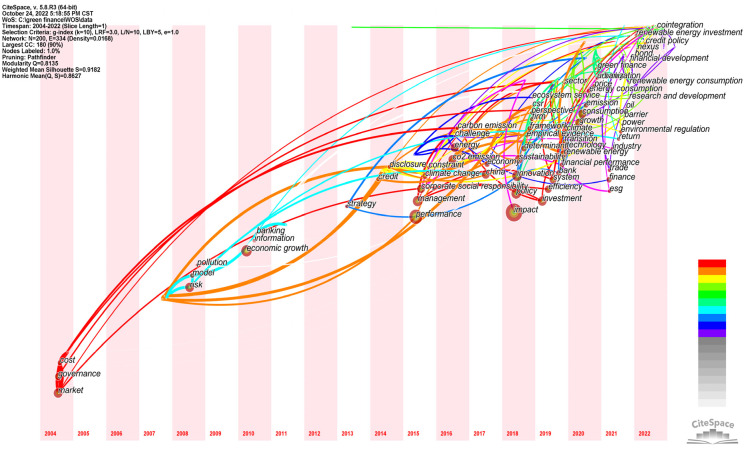
WOS time zone diagram.

Research hotspots are dynamic and vary in each time period. The CiteSpace software provides the Timezone View for the presentation of a literature co-citation network. By placing the keywords in the time zone where they first appear, a time series is arranged in the order from far to near, and then the concurrent area map of keywords is obtained by adjusting and embellishing. In this way, the research process of studying topics of interest in time dimension is clearly shown. On the basis of the keyword co-occurrence atlas, the time slice was set to 1 year and the other settings were kept unchanged. The “Run” button was clicked to obtain the keyword co-occurrence atlas, the “Layout” button was clicked on the quick control board of the visualization interface and “Timezone View” was selected under “Visualizations” to obtain the original atlas. By adjusting the parameters to further beautify the graph, we obtained the time zone chart of the domestic hotspots in the green finance research from 2000 to 2022, as shown in Figure 14. The time zone chart of the international research hotspots on green finance from 2004 to 2022 was also obtained, as shown in Figure 15.

Domestically, as shown in Figure 14, the node in the figure represents the keyword, and the year is when the keyword first appeared in the collected data (because the time slice was one year, it could only be determined as when the keyword first appeared in this year, and the specific year in which the keyword appeared in the statistical time range cannot be determined). If the number of occurrences is several, the circle will be larger accordingly. Therefore, the larger circle for the “green finance” node does not mean a higher occurrence frequency in the current year but the higher total occurrence frequency of the keyword in the collected data, indicating that the collected data were all centered on the discussion of green finance. The line represents the connection between the keywords, if the keywords appear in a paper at the same time, then there will be a line between the two keywords. If the two keywords appear in multiple papers at the same time, then the line is bold.

As shown in Figure 12 and Figure 14, in general, the domestic green finance research field has formed at a considerable scale, and the focus of green finance research has realized three stages of evolution, namely, in the early stage, it focused on green industries, such as photovoltaic power generation and low-carbon agriculture; in the middle stage, it focused on green services, such as green credit and green bonds; in the recent period, it focused on green strategies, such as green development, innovation and transformation. This indicates that domestic green finance research has undergone a spatial and temporal evolution from micro to macro and then to a long-term perspective. The research subjects have realized a diversified trend of focusing on enterprises in the early stage, institutions in the middle stage and government in the near future. The overall research direction is towards the creation of a high-quality green development environment. With the proposal of our innovation-driven development, high-quality development strategy, and the guidance of the five development concepts of innovation, coordination, green, openness and sharing, the current research focus is likely to continue for a period of time.

Internationally, as shown in Figure 15, the node in the figure represents the keyword. The year is the year in which the keyword first appeared in the collected data (because the time slice was 1 year, it can only be determined as the keyword’s first appearance in this year, and the specific year in which the keyword appeared in the statistical time range cannot be determined). The circle is larger accordingly, and the related lines represent the connection between the keywords. If the keywords appear in one paper at the same time, there is a line between the two keywords, and a connection between the two years will be generated. If the two keywords appear in multiple papers at the same time, the lines are bold.

As shown in Figure 13 and Figure 15, in general, “green finance” does not appear as a major node in the collected data, indicating that the international research on green finance is not only concerned with green finance but also the correlation between green finance and climate change, market players, government performance, sustainable economic and social development, social responsibility sharing and other factors, such as impact, performance, policy, management and sustainability. Corporate social responsibility has a bigger node. In addition, market, as the largest node appearing at the leftmost end of the time zone chart, indicates that international green finance research started from focusing on market entities. Governance, as the second largest node appearing at the leftmost end of the time zone chart, indicates that the international green research attaches great importance to the guiding role of the government. Both keywords, by focusing on market players and acknowledging the guiding role of the government, are aimed at reducing the cost of green development. For example, cost is another node with a high frequency at the leftmost end of the time zone chart in addition to market and governance.

#### 3.3.4. Keyword Emergent Analysis

“Emergent keywords” refer to keywords that are used with a sudden increase in frequency at a certain period of time, reflecting the importance of that certain keyword in that period of time. Chen Chaomei defines a research frontier as a group of emergent dynamic concepts and potential research questions that can accurately reflect the frontier fields of related disciplines. Emergent keywords are used to explore the emergent dynamic concepts and the potential research problems in the research on policy environment development; the causes behind them; reflect on the active or cutting-edge research nodes; and assist in predicting the areas of interest in the research and future trends. The basic principle of outburst word detection is that the frequency of a keyword variable surges over a short period of time and suddenly becomes a research hotspot, which can be understood as the “Baidu index” of academia. Since the emergent state of an emergent word usually has a time continuity, with a continuation period of 2 years or more, it can be used to assist in predicting research hotspots and trends in the future. At the same time, emergent word detection can be used to review which keywords became areas of interest in which time periods.

**Figure 16:**
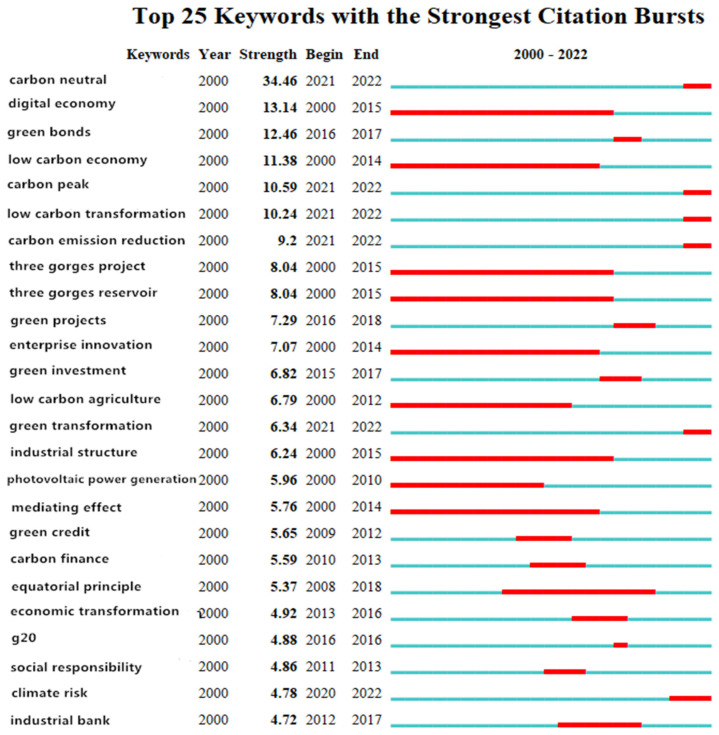
CNKI outburst chart.

**Figure 17:**
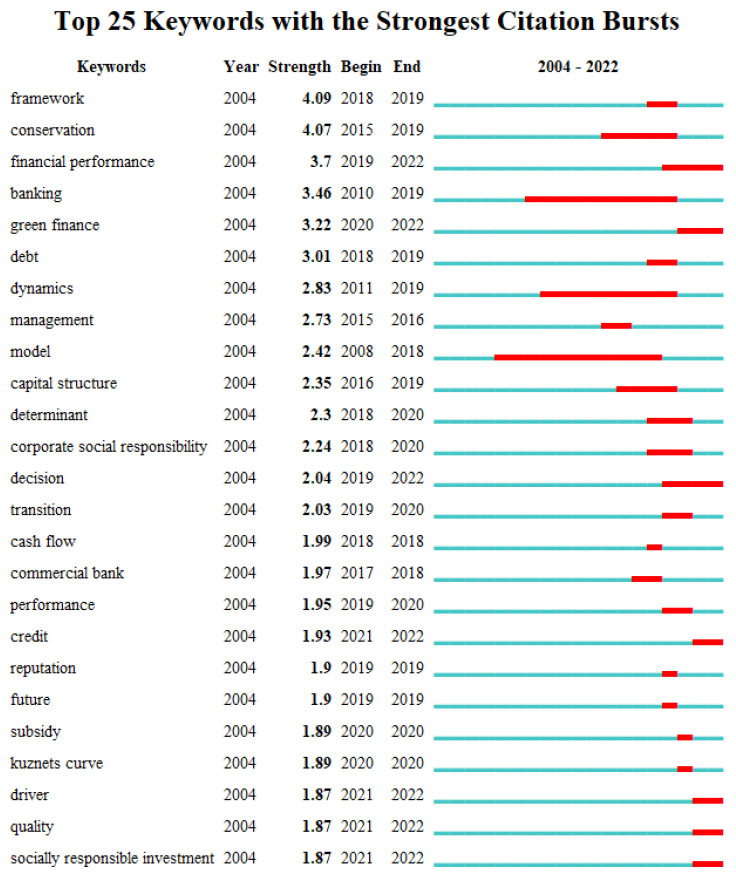
WOS outburst diagram.

In the case of the use of a Burmap for sudden keyword exploration, domestically, the γ value was set to 1, the parameter minimum duration was set to 1, and the following map was obtained (Figure 16). Internationally, the γ value was set to 0.6, the parameter minimum duration was set to 1, and the following graph was obtained (Figure 17). In the graph, “Begin” represents the beginning year of the keywords within the research time range, “End” represents the end year of the keywords, “Strength” represents the emergence intensity, the blue blocks represent the unit year time slice, and the red blocks represent the emergence period.

In Figure 16 and Figure 17, the keywords with a high emergence intensity in the CNKI green finance research included“carbon neutral”(34.46) “digital economy” (13.14), “green bonds” (12.46)““low-carbon economy” (11.38)”carbon peak”(10.59) and “low carbon transformation” (10.24), indicating that the above keywords were related to domestic research. In their corresponding period of attention at the forefront of the theme, the keywords with a high emergent intensity in the WOS policy environmental research were “framework” (4.09), “conservation” (4.07), “financial performance” (3.7), “banking” (3.46), “green finance” (3.22), “debt” (3.01), etc. This shows that the above keywords were the frontier topics that international researchers paid more attention to in their corresponding period. From the perspective of the time span of the outburst duration, the outburst duration of the CNKI policy environment research was relatively long: “digital economy”—16 years; “Three Gorges Project”—16 years; “Three Gorges Reservoir Area”—16 years; “industrial structure”—16 years; and “low-carbon economy”—15 years. These keywords have long been the concern of domestic scholars regarding the theme, and some are even topics of great interest. The emergence time of the WOS policy environment research lasted for a long time: “model”—11 years; “banking”—10 years; “dynamics”—9 years; “conservation”—5 years; and “capital structure”—4 years. This shows that these keywords have long been the subject of attention by international scholars. According to the time ranking, it can be found that the frontier keywords were constantly changing with the passage of time and showed a phased evolution. Therefore, this study classified the research frontiers in the field of policy environment according to the time stage, and it selected the keywords with highest emergent intensity in this period for analysis. From the domestic aspect, from 2000 to 2004 was the early stage, and the keywords with highest emergent intensity were “digital economy (13.14)”, “low-carbon economy” (11.38), “Three Gorges Project” (8.04), “Three Gorges Reservoir Area” (8.04) and “enterprise innovation” (7.07). This shows that the focus of attention of researchers in this period was to stimulate the new driving force of green development and to strengthen the support of green development. The period from 2005 to 2015 was the middle period, and the keywords with the highest emergent intensity were “green investment” (6.82), “green credit” (5.65) and “carbon finance” (5.59), indicating that the areas of interest by researchers during this period were focused on green financial products, such as green investment, green credit and carbon finance. From 2016 to 2022, the keywords with highest emergent intensity were “carbon neutrality” (34.46), “carbon peak” (10.59) and “low-carbon transition” (10.24). This indicates that the focus of attention of researchers in this period was on green, low-carbon and high-quality development strategies and transformation goals, such as carbon peak, carbon neutrality and low-carbon transition. Internationally, the period from 2004 to 2011 was the early stage, and the keywords with the highest emergent intensity were “banking” (3.46), “dynamics” (2.83), “model” (2.42) and “management” (2.73), indicating that the international areas of interest in this period were concentrated in “banking”, “driving force”, “model” and “management”. In the middle period from 2012 to 2019, the keywords with the highest emergent intensity included “framework” (4.09), “conservation” (4.07) and “financial performance” (3.7). This shows that the international research focus in this period was to improve the green finance system and strengthen the performance evaluation of green finance. From 2020 to 2022, the keywords with the highest intensity in subsidy were “green finance” (3.22), “credit” (1.93) and “Kuznets curve” (1.89). This shows that the focus of international research during this period was on the coordination of green finance, such as finance, credit and bonus. In the next few years, the research frontiers of green finance in China included climate risk, carbon neutrality, carbon peaking, low-carbon transition, carbon emission reduction and green transition. The international research frontiers on green finance included financial performance, decision, green finance, credit, driver, quality and socially responsible investment.

## 4. Analysis of Policy Texts

The law of rooted theory is often used to explore fuzzy concepts. In fact, in recent years, with the development of green finance in our country, the connotation and extension of green finance in our country has been very clear, but from the perspective of policy science, green finance policy as a system policy, how it works, by which policy elements and how they are combined are still very unclear. Therefore, in this section, we collected green finance policy texts on the basis of CiteSpace software’s keyword and cluster analysis of the green finance research literature and drew on grounded theory for the text analysis.

### 4.1. Collection of Legal and Policy Texts

Law and policy are two complementary means of modern social regulation and governance. A legal text is the carrier of legal norms and the product of legislators’ intentions expressed through text. A policy text is a clear expression of actionable principles, objectives and tasks; the steps and measures taken by the government; and the relevant social subjects to implement relevant activities in a certain period of time. It reflects the intention of decision-making departments to guide relevant social activities, and the governance intention of rulers can be found by analyzing legal and policy texts. To this end, this study used tools such as the Chinese government (www.gov.cn, accessed on 16 October 2022), Peking University Talisman and CNKI to retrieve and collect 22 legal and policy texts related to green finance (as shown in Table 6). Among them, 19 policy texts were selected for coding analysis and model construction, and the remaining 3 were used for saturation testing of grounded theories. (The legal and policy texts are shown in Table 6).

### 4.2. Analysis of the Legal and Policy Texts

In this paper, based on the grounded theory method, the content extracted from the above 22 legal and policy texts was conducted according to a three-level coding in an attempt to sort out the specific requirements of the laws and policies on green finance.

#### 4.2.1. Open Coding

Open coding is the process of interpreting disordered raw data in order to discover new insights and generate initial concepts from the phenomena presented by the data [56]. In this paper, the 938 selected original sentences were initially conceptualized by labeling them sentence by sentence. In order to reduce the subjective factors of the researchers, the original words of the legal and policy texts were used as the code of the labels as much as possible. The concepts whose frequency was less than three and could not be categorized were eliminated, and 30 initial categories were finally formed. They were propaganda leads (9, reference points, same below); practical issues (3); implementing the decisions and arrangements of superiors (18); supporting green, low-carbon and high-quality development (13); promoting the development of green finance (12); serving economic activities with both environmental and social benefits (7); promoting the transformation and upgrading of the economic structure and the transformation of the economic development mode (7); practicing the concept of green development (5); promoting carbon peak and carbon neutrality (5); addressing climate change (3); product development (393); risk management (56); technological governance (43); international connection (32); pilot demonstration (31); industrial and industrial guidance (26); standard system (25); improving mechanisms (22); capacity building (19); supporting local governments (15); market operation (13); connecting society (10); cooperation promotion (3); policy implementation (3); supervision and management (68); information disclosure (51); incentives and constraints (40); third-party support (26); accountability according to regulations (16); and revision and improvement (4), as shown in Table 7 (being limited by space, only the partial results are listed).

#### 4.2.2. Axial Coding

The main task of axial coding is to extract the relationship between categories and the categories according to the coding mode. It is a complex inductive deduction process that connects subcategories and main categories involving multiple steps [57,58]. Through axial coding, this paper further summarized the above 30 categories into 17 subcategories, policy beliefs and policy objectives, including problem belief, value belief, mission, vision, values, regulatory tools, support service tools, market tools, test tools, innovation tools, disclosure feedback, regulatory feedback, guidance feedback, accountability feedback, third-party feedback, revision cycle and cohesion cycle. There were five main categories, including standard, policy instrument, policy feedback and policy cycle, as shown in Table 8.

#### 4.2.3. Selective Encoding

The purpose of selective coding is to discover a core category from a main category, and establish the relationship between the core category and other categories in the way of a story line so as to refine the theoretical model of the research [59]. This is the final step in the coding analysis and formation of a theoretical framework. The core category determined in this study was “governance logic of China’s green finance policy”, which consisted of five main categories: policy belief, policy objectives, policy tools, policy feedback and policy cycle.

The story line surrounding this core category can be roughly understood as follows: the governance logic of China’s green finance policy is a dynamic process driven by policy beliefs. From Sabatier’s division and definition of a belief system, a belief system can be regarded as a set of basic values, a causal hypothesis and the resulting cognitive system of problems. Policy core beliefs refer to the basic strategy and fundamental policy position concerning obtaining deep core beliefs in the policy domain or subsystem. For our country, different levels of government will differ in the instrumental belief in the secondary aspects of the goal of achieving the core values of the policy, but the core beliefs of the policy are the same. It is precisely because of the consistency of core policy beliefs that governments at all levels can get together and coordinate their actions, thus leading to the emergence of policy interaction. To put it simply, a policy goal is the policy vision with a clear outline reached by governments at all levels under the driver of policy belief. It is the derivative of a policy belief and the action guide of other policy governance links in addition to a policy belief. A policy tool is the specific policy means adopted by governments at all levels to promote the realization of policy objectives. It is the selection and integration process of different types of policy tools, such as regulation, supply and consumption. Policy feedback is the process of publicly feeding back the policy results generated by the operation of policy tools to governments at all levels and implementing rewards and punishments. The purpose of performance feedback is to let the evaluated object (at all levels of government) know whether the predetermined goals have been achieved, and then making improvement plans. Policy cycle is an activity and process that is based on policy feedback and combined with new policy information to help policy subjects to gradually adjust and improve policy tools to achieve policy objectives.

Specifically, the story line produced by our country’s green financial policy governance process should be expressed as: Under the background of a warming climate and a severe environmental situation, the policy subject realizes the importance of constructing and improving the green finance governance system to serve the urgency of giving consideration to both economic development and environmental protection economic activities. Then, through a series of propaganda and educational activities that are required by some policies, the policy implementation subject object reaches a consensus on developing green finance in our country. Based on this, the concept of green development can be established and practiced, green transformation development can be promoted and, finally, the policy goals of “carbon peak” and “carbon neutrality” can be achieved. Then, a mixture of regulation, support services, market, experiment, innovation and other policy tools can be used, and various policy feedback forms, such as disclosure, supervision, guidance and third-party feedback, can be used to continuously achieve policy goals and consolidate policy beliefs. At the same time, with the development of green finance practice, the policy system of green finance is constantly revised and improved, and the activities and processes of connecting and coordinating with policies in energy, environmental protection, economic and social development and other fields can be conducted.

**Figure 18:**
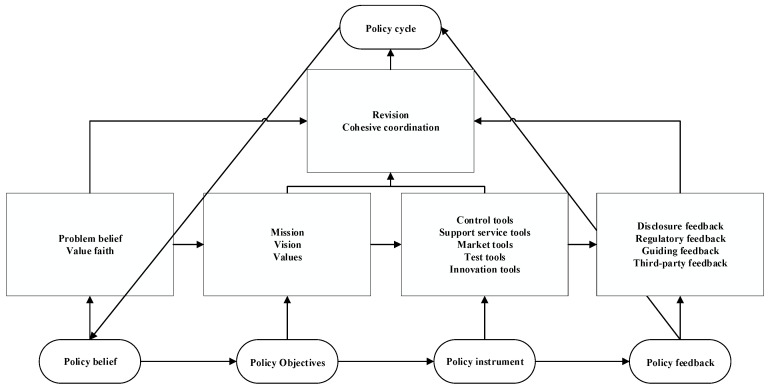
China’s green finance policy governance model.

Based on the above analysis, China’s green finance policy governance model can be constructed as per below (see Figure 18).

Based on the above construction model, combined with CiteSpace’s hotspot and cluster analysis, this study reviewed texts on green financial policy and found the following problems in our green financial policy: first, it is difficult to innovate the transmission path of policy tools and the insufficient promotion of policy; second, the feedback on regulatory policies was the most, while the feedback on soft policies was insufficient; third, China’s green finance policies are seriously fragmented, with inadequate policy circulation and cohesion.

#### 4.2.4. Theoretical Saturation Test

A theoretical saturation test means that when no new attributes of a category derived from theoretical sampling data emerge, the attributes of the category are saturated and no new properties of the theoretical categories emerge; thus, theoretical saturation has been reached [60]. This study also conducted a coding analysis of the remaining three policy texts according to grounded theory procedure to test the theoretical saturation. The results show that the categories overlapped and resembled each other, and no new important categories and relations appeared. The categories in the model were quite abundant. Therefore, it can be considered that the above green finance policy governance process model is theoretically saturated.

## 5. Conclusions

This paper mainly consisted of two parts of research content: One was the use of CiteSpace software to conduct a visual comparative analysis of green financial knowledge ecology at home and abroad. Second, NVivo12plus software was used to root and code the existing 22 green finance research policy texts in China.

The results of the visualization study show the following:

(1) The number of “green finance” studies at home and abroad presents a wave rising trend. Both home and abroad attach great importance to the study of green finance, and the study of green financialization has broad prospects.
(2) The core authors of domestic green finance research have initially formed, while the core authors of international green finance research have not yet formed.
(3) There is a lack of close cooperation between domestic institutions with important contributions to the field of green finance, and the degree of communication is relatively low. Compared with domestic institutions, the number of publications of international influential institutions is less, but international influential institutions are closely connected, and a certain scale of cooperation network has formed.
(4) The influential countries in the field of green finance are Singapore, France, Switzerland, Canada and Saudi Arabia.
(5) From the perspective of the focus of green finance research, both domestic and international countries attach great importance to balancing the relationship between economic growth and a low-carbon green transition. However, Chinese green finance research focuses more on macroeconomic development and strategic transition, while the international community tends toward microcorporate green performance, policy optimization and market innovation.
(6) From the perspective of the characteristics of the research hotspots, domestic policy environment research areas of interest focus on the thorny issues facing social and economic development, close to social practice but not enough prospective and guiding research on social practice. The international emphasis on the theoretical model of the practice of policy environment optimization of the theoretical guidance is stronger.
(7) From the perspective of the evolution trends in this study, the domestic green finance research field has formed at a considerable scale, and the focus of green finance research has achieved three stages of evolution: in the early stage, it focuses on green industries, such as photovoltaic power generation and low-carbon agriculture; in the middle stage, it focuses on green services, such as green credit and green bonds; and in the recent period, it focuses on green strategies, such as green development, innovation and transformation. This indicates that domestic green finance research has undergone a transformation from micro to macro and then to a long-term spatial and temporal perspective. Researchers have realized a diversified trend from focusing on enterprises to focusing on institutions and then to focusing on the government. The overall research direction is to create a high-quality green development environment. With the introduction of the strategy of innovation-driven development and high-quality development and the guidance of the five development concepts of innovation, coordination, green, opening up and sharing, the current research focus is likely to continue for a while. International research on green finance focuses on climate change, market players, government performance, sustainable development of economy and society, sharing of social responsibilities, etc. In particular, the international green research field attaches great importance to the market and the guiding role of government. Reducing the cost of green development is topic of great interest in international green finance.
(8) In terms of research frontiers, the domestic research frontiers on green finance include climate risk, carbon neutrality, carbon peaking, low-carbon transition, carbon emission reduction and green transition. The international research frontiers on green finance include financial performance, decision, green finance, credit, driver, quality and socially responsible investment.

The results of the root coding study show the following:

(1) Policy belief is the logical starting point of China’s green finance policy governance process, and it plays an ideologically leading role in the whole process of green finance policy governance under the influence of an external environmental situation. Policy belief consists of problem belief and value belief.
(2) Policy objectives are the mission, vision and values of China’s green finance policy development. Driven by policy beliefs, it is a set of specific missions, visions and values that can be measured and evaluated qualitatively or quantitatively. It is the action guide and main follower of subsequent policy links. Policy goals consist of mission, vision and values. The mission is the responsibility of the subject and the object of the policy in dealing with climate change and promoting green and high-quality development. It affects the direction of the policy and directly determines the direction of the policy vision.
(3) Policy tool is a combination of policy means adopted by the government to achieve policy objectives. It is the most critical link in the process of China’s green finance policy governance, because it is the process in which the government implements policy objectives with policy beliefs, and it is also the key reference link in the following policy links. The process of its actual occurrence is the process of testing the enforceability of a policy text, the process of testing whether policy implementers are firm in their policy beliefs, and the process of testing the complexity of the interest demands of different policy subjects. It is the most active, uncertain and complicated key link in the whole process of China’s green finance policy governance.
(4) Policy feedback is an important part of China’s green finance policy governance process, which is of great significance for improving policy quality and promoting policy coordination. It mainly consists of four categories: disclosure feedback, supervision feedback, guidance feedback and third-party feedback. It will escort the realization of policy objectives and improve the effectiveness of policy tools.
(5) The policy cycle is an activity and process in which the subject and object of policy adjust, improve, perfect, revise and connect policies according to policy tools and policy feedback results in order to achieve policy goals under the guidance of policy beliefs. It is the inflection point of the closed-loop management of green finance policy within a certain policy time limit. Therefore, it is not only the end point but also the starting point, or the link that is always associated with policy beliefs, policy objectives, policy tools, policy feedback and other policy links. It is a progressive revision and improvement process, showing a spiraling trend. It is also a very important link which is poorly governed and prone to policy circle. The so-called policy circle is to recycle policy texts with no substantial improvement to the content, which makes policy governance become the use of policy texts to govern policy texts and even leads to the illusion of policy belief, the loss of policy objectives, the contradiction and disorder of policy tools and the obstruction of policy cycle. The whole policy governance process is made into formalism. Green finance policies involve various fields, such as environment, energy, economy and social development. How to change the fragmentation of China’s green finance policies and how to achieve policy cohesion is also a very key issue.

## 6. Countermeasures and Suggestions

Through visual analysis and rooted coding research, this study drew the following policy suggestions that are conducive to the development and improvement of China’s green finance policy.

### 6.1. Guide the Main Body of Green Finance Policy to Establish Firm Policy Beliefs

On the one hand, green finance policies involve a large number of subjects with different interest demands, and the risks are relatively large if the policy beliefs are not firm. On the other hand, the belief of green finance policy requires higher value rationality and problem rationality of policy subjects, and it is difficult to publicize and guide them. Nowadays, the radiation of green finance policy in our country mainly consists of the large financial institutions and enterprises, and the radiation of small and medium-sized enterprises and consumers is not enough. Therefore, it is necessary to strengthen the guidance of policy beliefs on green finance policy subjects, give full play to the ecological creation function of major media platforms through various emerging digital technology means, such as we media and short videos, closely track the climate and environmental change situation and its impact on human society, timely convey effective information and maximize the coverage of all policy subjects. All policy subjects should be guided to firmly promote the development of green finance belief so that it can better support and serve the green transformation of economic activities.

### 6.2. Improve the Clarity of Green Finance Policy Objectives

The main manifestation of the difference between green finance policy and policy belief is whether the goal of green finance policy is clear and whether it can be evaluated quantitatively. Policy objectives are the extension of policy beliefs, the direction of policy tools and the basis of policy feedback. Whether the policy objectives are clear directly affects the operational quality of other policy links. Affected by the limits of policy implementation, the main body of grassroots policy has a tendency to pursue the fuzzy policy objectives. Therefore, to improve the governance capacity of green finance policy, it is necessary to strengthen the top-level design, improve the clarity of policy objectives, adhere to the principle of quantitative first and qualitative second and a combination of qualitative and quantitative, divide quantitative indicators into time schedules, anchor qualitative indicators as specific matters, and make policy objectives visible, tangible, verifiable and measurable, in addition to promoting the implementation of policy objectives with high clarity.

### 6.3. Enhance the Overall Governance Effectiveness of Green Finance Policy Instruments

On the one hand, the product forms of our green financial policy tools are mainly green credit, green bond and green fund. The form of green financial policy tools is single, which seriously affects the efficiency of green financial policy tools. On the other hand, the innovation capacity of green financial policy instruments is insufficient, the digitalization of green finance is still in its infancy and the fragmentation of green financial policy governance is prominent; governance efficiency needs to be improved. Therefore, it is necessary to adhere to the concept of holistic governance, strengthen top-level design, apply systematic thinking, make good use of a variety of policy tools, improve the coordination of all policy tools and enhance the overall governance effectiveness of green finance policy tools.

### 6.4. Strengthen the Construction of the Green Finance Incentive Policy Feedback System

At present, our policy feedback link mainly focuses on supervision and management. Although there are many incentive and reward contents, such as “encouragement” and “support”, in the policy texts, it mostly stays in the expression of the government’s attitude and has insufficient attraction to the actual subject of the policy. Therefore, it is necessary to use a mixture of fiscal, monetary, tax and other policy means, give full play to the important role of the third party in the green finance policy feedback link, innovate service forms and methods and constantly strengthen the construction of the green finance incentive policy feedback system. There should be a further increase in terms of information disclosure on green finance policies, the establishment of a positive and negative list system for green finance, especially for increasing the disclosure of the beneficiaries of green finance incentive policies, and effective improvement of the positive feedback effect of green finance policies.

### 6.5. Improve the Quality of Green Finance Policy Circulation

In other words, green financial policies have higher requirements for policy sensitivity, and the tension between policy stability and policy sensitivity has higher requirements for the cycle of green financial policies. On the one hand, at present, the quality of China’s green finance policy cycle is relatively poor. The existing revisions and improvements mostly depend on the potential guidance of higher-level decisions and deployment, mostly due to the small scope adjustment of policy texts and the insufficient local characteristics. On the other hand, the degree of cohesion of the policies is not high enough to linkage various fields, such as environment, economy, energy, society and corporate governance. Therefore, we should strengthen the top-level design, coordinate the revision, improve and make cohesive the tools of green finance policy at a central level, strive to draw a blueprint that reaches an end, promote the innovation of Chinese ESG and effectively improve the quality of our green finance policy.

### 6.6. Research Limitations and Prospects

First, this study took authoritative theoretical literature and the policy texts on green finance as research samples to study the knowledge ecology and policy governance logic of green finance in China. It has a certain representativeness and restriction because it reflects the knowledge picture of green finance in China and the top-level design of policy. The limitation lies in that the selected research samples were second-hand materials. First-hand materials obtained from non-field studies describe the overall macrolevel development of green finance policy, and the implementation of the policy in local areas cannot be investigated. Therefore, the theoretical guidance of green finance policy research is too guiding, and the practical exploration is insufficient.

Secondly, this study adopted qualitative research methods in the study of policy texts. Although the coding and theoretical construction were carried out in strict accordance with the steps of grounded theory, the subjectivity of the research cannot be avoided. In the future, program software can be used to conduct more scientific coding and theoretical construction, and a quantitative analysis method can be adopted to verify and improve the model.

Finally, most of the references in this study are local to China, which reflect the localized governance status of green finance in China. The research conclusions have certain limitations and limited inspiration for global green finance policy governance.

## Figures and Tables

**Table 1:** Comparison of the number of publications by the authors (top 10).

Serial Number	Author	Count	Year	Author	Count	Year
1	Jun Ma	17	2016	Farhad Taghizadehhesary	15	2019
2	Guojun An	17	2016	Zhen Liu	6	2022
3	Shangqi Liu	16	2000	Sangbing Tsai	6	2018
4	Haoming Shi	15	2000	Muhammad Mohsin	6	2021
5	Yahong Zhou	13	2000	Abu Bakkar Siddik	5	2021
6	Teng Zhou	11	2000	Naoyuki Yoshino	5	2019
7	Yao Wang	11	2015	Muhammad Abubakr Naeem	5	2021
8	Chenying Zhou	11	2000	Kangyin Dong	5	2022
9	Qi Fang	10	2019	Martina K Linnenluecke	4	2016
10	Lihua Qian	9	2017	Fengsheng Chien	4	2021

**Table 2:** Comparison of the number of publications by research institutions (top 10).

CNKI (Top 10)			WOS (Top 10)		
Institution	Count	Year	Institution	Count	Year
School of Economics, Xihua University	43	2000	Jiangsu University	19	2020
Institute of Finance and Banking, Chinese Academy of Social Sciences	32	2016	Capital University of Economics and Business	15	2018
Green Finance Committee, China Society for Finance and Banking	29	2016	Tokai University	13	2020
School of Economics, Shanghai University of Finance and Economics	20	2000	Cent University of Finance and Economics	12	2021
Chengdu Branch of the People’s Bank of China	18	2000	Anhui University of Finance and Economics	11	2021
School of Agriculture and Rural Development, Renmin University of China	17	2000	University of Oxford	11	2012
Industrial Bank	17	2016	Southwestern University of Finance and Economics	11	2021
Institute of Finance and Banking, People’s Bank of China	16	2017	China University of Mining and Technology	10	2019
Research Bureau of the People’s Bank of China	16	2016	University of International Business and Economics	10	2022
School of Management, Donghua University	16	2000	Beijing Institute of Technology	10	2018

**Table 3:** Comparison of the number and centrality of national publications.

	Count (Top 10)			Centrality (Top 10)		
Serial Number	Country	Count	Year	Country	Centrality	Year
1	People’s Republic of China	417	2008	Singapore	0.91	2010
2	United Kingdom	70	2012	France	0.51	2010
3	United States	61	2007	Switzerland	0.4	2007
4	Italy	54	2012	Canada	0.35	2016
5	Pakistan	51	2018	Saudi Arabia	0.32	2019
6	Australia	45	2008	Australia	0.3	2008
7	France	40	2010	United States	0.26	2007
8	Germany	36	2017	Italy	0.25	2012
9	Malaysia	33	2018	Japan	0.25	2019
10	Spain	31	2018	United Kingdom	0.23	2012

**Table 4:** Keyword frequency comparison table.

CNKI (Top 10)				WOS (Top 10)			
Keyword	Count	Centrality	Year	Keyword	Count	Centrality	Year
Green finance	1021	0.36	2000	Impact	129	0.19	2018
Green credit	267	0.81	2008	Performance	95	0.2	2015
Green bonds	188	0.03	2014	Investment	80	0.15	2019
Carbon neutral	102	0.19	2020	Economic growth	76	0.29	2010
Green development	95	0.06	2016	Policy	70	0.04	2018
Digital economy	48	0.47	2000	CO_2_ emissions	60	0.3	2016
Rural revitalization	48	0.08	2000	Growth	54	0.03	2020
Green industry	45	0.34	2002	Management	53	0.13	2015
Low-carbon economy	45	0.25	2000	Innovation	52	0.17	2018
Carbon finance	42	0.1	2010	Market	49	0.09	2004

**Table 5:** Clustering summary and comparison.

Database	Label	Number of Nodes	Value of Contour	Year	Keywords
CNKI	0	25	0.977	2012	Green credit (90.98, 1.0 × 10^−4^); commercial bank (72.8, 1.0 × 10^−4^); equator principle (33.75, 1.0 × 10^−4^); industrial bank (24.44, 1.0 × 10^−4^); finance (19.8, 1.0 × 10^−4^)
	1	23	1	2011	Green finance (140.11, 1.0 × 10^−4^); wind power generation (25.76, 1.0 × 10^−4^); subsidy “backsliding” (25.76, 1.0 × 10^−4^); photovoltaic power generation (25.76, 1.0 × 10^−4^); panel vector autoregression (24.81, 1.0 × 10^−4^)
	2	16	1	2016	Green bond (103.47, 1.0 × 10^−4^); investors (65.63, 1.0 × 10^−4^); issuer (56.21, 1.0 × 10^−4^); information disclosure (44.39, 1.0 × 10^−4^); fund Raising (22.44, 1.0 × 10^−4^)
	3	16	0.877	2015	Industrial structure (105.33, 1.0 × 10^−4^); energy utilization (99.78, 1.0 × 10^−4^); digital economy (93.09, 1.0 × 10^−4^); resource allocation (91.79, 1.0 × 10^−4^); low-carbon economy (65.56, 1.0 × 10^−4^)
	4	15	0.911	2015	Green industry (42.17, 1.0 × 10^−4^); financing constraints (38.24, 1.0 × 10^−4^); financial development (23.63, 1.0 × 10^−4^); environmental regulation (23.4, 1.0 × 10^−4^); green finance development (19.07, 1.0 × 10^−4^)
	5	15	0.854	2016	Green fund (29.37, 1.0 × 10^−4^); green financing (21.72, 1.0 × 10^−4^); banking industry (19.06, 1.0 × 10^−4^); standard system (15.81, 1.0 × 10^−4^); institutional mechanisms (15.81, 1.0 × 10^−4^)
	6	14	0.976	2018	Financial inclusion (67.86, 1.0 × 10^−4^); financial innovation (30.2, 1.0 × 10^−4^); common prosperity (21.05, 1.0 × 10^−4^); prevention and resolution (19.46, 1.0 × 10^−4^); agricultural supply side reform (12.96, 0.001)
	7	13	0.982	2018	Climate change (40.89, 1.0 × 10^−4^); climate risk (40.79, 1.0 × 10^−4^); pressure test (21.58, 1.0 × 10^−4^); ecological civilization (21.24, 1.0 × 10^−4^); financial stability (14.97, 0.001)
	8	12	0.923	2021	High-carbon industry (41.84, 1.0 × 10^−4^); low-carbon transition (30.64, 1.0 × 10^−4^); addressing climate change (16.32, 1.0 × 10^−4^); transformation finance (15.9, 1.0 × 10^−4^); category of contents (13.74, 0.001)
	9	12	0.987	2019	Carbon neutral (75.61, 1.0 × 10^−4^); carbon peaked (73.36, 1.0 × 10^−4^); Green transformation (25.73, 1.0 × 10^−4^); carbon emission reduction (23.38, 1.0 × 10^−4^); driving factors (16.58, 1.0 × 10^−4^)
WOS	0	21	0.905	2015	Climate risk (7.48, 0.01); energy policy (7.48, 0.01); influencing factors (7.07, 0.01); co2 emissions (5.31, 0.05); carbon emissions (4.64, 0.05)
	1	20	0.933	2016	Carbon emission (8.22, 0.005); gmm (7.72, 0.01); Bangladesh bank (7.72, 0.01); green banking (6.84, 0.01); co2 emission (5.62, 0.05)
	2	17	0.974	2016	Difference-in-difference model (6.2, 0.05); green enterprises (6.2, 0.05); cross listing (6.2, 0.05); green logistics (6.2, 0.05); retrofit (6.2, 0.05)
	3	16	0.749	2020	Renewable energy (5.62, 0.05); rebranding (5.02, 0.05); environment management (5.02, 0.05); innovation diffusion (5.02, 0.05); wind energy (5.02, 0.05)
	4	15	0.858	2022	Ols (14.5, 0.001); top 10 co2 emitting countries (10.29, 0.005); public spending (10.29, 0.005); green energy finance (10.29, 0.005); DEA (7.49, 0.01)
	5	14	0.966	2019	Mechanism (9.8, 0.005); financial constraint (6.17, 0.05); firm performance (5.03, 0.05); carbon financial products (4.9, 0.05); competitiveness (4.9, 0.05)
	6	13	0.976	2011	Emissions markets (10.01, 0.005); cap-and-trade schemes (10.01, 0.005); asset pricing (10.01, 0.005); investment strategies (10.01, 0.005); sustainability indices (6.36, 0.05)
	7	11	0.978	2016	Risk management (6.75, 0.01); sufferers (6.04, 0.05); collusion (6.04, 0.05); coping (6.04, 0.05); EU law (6.04, 0.05)
	8	11	0.905	2018	Blockchain (11.92, 0.001); porter hypothesis (6.58, 0.05); Gobernanke za (5.95, 0.05); protected area (5.95, 0.05); cluster analysis (5.95, 0.05)
	9	10	0.928	2020	co2 emissions (12.29, 0.001); financial development (11.12, 0.001); ecological footprint (7.06, 0.01); input–output model (6.2, 0.05); nitrous oxide (6.2, 0.05)

**Table 6:** List of legal and policy texts.

Serial Number	Symbol Number/Date	Title
N01	CBIRC 15 (2022)	Notice of China Banking and Insurance Regulatory Commission on Issuing Green Finance Guidelines for the Banking and Insurance Industries
N02	27 May 2021	Notice of the People’s Bank of China on the Printing and Distributing of the Green Finance Evaluation Plan for Banking Financial Institutions
N03	PBC 29 (2018)	Notice of the People’s Bank of China on Matters Concerning Strengthening the Supervision and Administration of the Duration of Green Finance Bonds
N04	31 August 2016	The People’s Bank of China, the Ministry of Finance, the Development and Reform Commission and Other Guidelines on Building a Green Finance System
N05	PBC 180 (2022)	Notice of the People’s Bank of China, the Development and Reform Commission, the Ministry of Finance, the Ministry of Ecology and Environment, the Banking and Insurance Regulatory Commission and the Securities Regulatory Commission on Printing and Distributing the Overall Plan for Building a Green Finance Reform and Innovation Pilot Zone in Chongqing
N06	PBC 116 (2019)	Notice of the People’s Bank of China on Supporting the Issuance of Green Debt Financing Instruments in the Green Finance Reform and Innovation Pilot Zone
N07	Bulletin PBC No. 39 (2015)	Announcement of the People’s Bank of China (2015) No. 39—Announcement on Matters Related to the Issuance of Green Finance Bonds in the Interbank Bond Market
N08	NAFMII No. 7 (2022)	The National Association of Financial Market Institutional Investors on Strengthening the Self-discipline Management of Green Finance Bond Duration Information Disclosure
N09	CBRC Office No. 186 (2014)	Notice of the General Office of the China Banking Regulatory Commission on Printing and Distributing the Key Evaluation Indicators for the Implementation of Green Credit
N10	CBRC No. 4 (2012)	Notice of the China Banking Regulatory Commission on Issuing Green Credit Guidelines
N11	CBRC Office No. 40 (2013)	Opinions of the CBRC General Office on Green Credit Work
N12	Announcement of the People’s Bank of China and China Securities Regulatory Commission No. 20 (2017)	Announcement of the People’s Bank of China and the China Securities Regulatory Commission (2017) No. 20—Guidelines on the Evaluation and Certification of Green Bonds (Interim)
N13	Announcement of the China Securities Regulatory Commission No. 6 (2017)	The China Securities Regulatory Commission’s Guidance on Supporting the Development of Green Bonds
N14	PBC 96 (2021)	Notice of the People’s Bank of China, the National Development and Reform Commission and the China Securities Regulatory Commission on Issuing the Catalogue of Projects Supported by Green Bonds (2021 Edition)
N15	Development and Reform Office Finance No. 3504 (2015)	Notice of the General Office of the National Development and Reform Commission on Issuing Guidelines on the Issuance of Green Bonds
N16	China Municipal Cooperative Development No. 172 (2022)	Notice of the China Interbank Market Dealers Association on Matters Related to the Evaluation and Certification Institutions’ Operation of Green Debt Financing Instruments
N17	Bulletin NAFMII No. 10 (2017)	Announcement of the Association of China Interbank Market Traders No. 10 (2017)—Announcement on the Release of the Guidance on Green Debt Financing Instruments for Non-Financial Enterprises and Supporting Forms
N18	JR/T 0227-2021	Guidelines on the Environmental Information Disclosure for Financial Institutions
N19	JR/T 0228-2021	Environmental Rights Financing Vehicle
N20	MEEC Climate 57 (2020)	The Ministry of Ecology and Environment, the National Development and Reform Commission, the People’s Bank of China and other guidelines on Promoting Investment and Financing in Response to Climate Change
N21	26 April 2021	General Offices of the CPC Central Committee and The State Council Issue Opinions on Establishing and Improving the Value Realization Mechanism of Ecological Products
N22	Development and Transformation Energy No. 206 (2022)	Opinions of the National Development and Reform Commission and the National Energy Administration on Improving the Institutions, Mechanisms, Policies and Measures for the Green and Low-Carbon Energy Transition

**Table 7:** Open coding (partial).

Category (Reference Point/Piece)	Original Representative Statement
Promoting the development of green finance (12)	Promoting the development of the banking and insurance industry (N01)
Tackling climate change (3)	Better leverage of the supporting role of investment and financing in addressing climate change (N20)
Product development (393)	Market investment institutions will be encouraged to develop public and private funds and other green financial products based on the Green Index to meet the needs of investors (N13)
Risk management (56)	Banking and insurance institutions shall urge their clients to strengthen environmental, social and governance risk management by improving contract terms (N02)
Docking international (32)	Promoting international cooperation in green finance (N04)
Pilot demonstration (31)	Adhere to the first trial; the risk is controllable (N05)

**Table 8:** Axial coding.

Category of Principal	Secondary Category	Category of Influence Relationship	Connotation of Relationship
Policy belief	Problem belief Belief in value	A real problem	Policy beliefs include perceptions of important causal relationships and perceptions of the importance of the problem This is the logical starting point of green financial policy governance
		Publicity and guidance	
Policy objectives	Mission Vision Values	Tackling climate change	Beliefs derive goals. Under the guidance of policy beliefs, governments at all levels generate a sense of mission, reach a common vision, decompose the vision into values, integrate values into development strategies, and embody sub-goals. It is the spiritual link of green finance policy governance logic
		We will support green, low-carbon and high-quality development	
		Promote carbon peak and carbon neutral	
		To implement the decisions and arrangements made by superiors	
		We will promote green finance	
		To serve economic activities with both environmental and social benefits	
		We will promote economic structural transformation and upgrading and change the pattern of economic development	
		Practicing green development	
Policy instrument	Instrument of control Support service tools Tools of market Tools of test Tools for innovation	System of standards	Policy tools are the process in which governments at all levels adopt a series of policy measures and mobilize various resources to organize, coordinate and control in order to turn the vision into reality. They are the key links of green finance policy governance
		Improve the mechanics	
		Implementation of policy	
		Risk management	
		International connection	
		Promote cooperation	
		Support places	
		Connected society	
		Capacity building	
		Industry and industry guidance	
		Product development	
		Operation of market	
		Pilot demonstration	
		Technical management	
Policy feedback	Disclosure of feedback	Disclosure of information	Policy feedback is the activity and process of correcting and optimizing policy tools, ensuring the realization of policy objectives and an important link of green finance policy governance
	Regulatory feedback	Supervision and administration	
	Guide feedback	Incentive and constraint	
	Feedback on accountability	Accountability according to regulations	
	Third-party feedback	Third-party support	
Policy cycle	Cycle of revision	Revision and improvement	Policy cycle is the process of policy improvement and revision under the influence and promotion of policy feedback results. This is an indispensable link of green finance policy governance. It is not only the last link of this round of policy governance but also a new starting point for progressive improvement of governance, that is, the beginning of the next round of policy governance
	Fit in		

## Data Availability

The data used to support the findings of this study are available from the corresponding author upon request.
